# Deep learning-based detection algorithm for brain metastases on black blood imaging

**DOI:** 10.1038/s41598-022-23687-8

**Published:** 2022-11-14

**Authors:** Jang-Hoon Oh, Kyung Mi Lee, Hyug-Gi Kim, Jeong Taek Yoon, Eui Jong Kim

**Affiliations:** 1 Department of Biomedical Science and Technology, Graduate School, Kyung Hee University, #23 Kyungheedae-Ro, Dongdaemun-Gu, Seoul, 02447 Republic of Korea; 2 Department of Radiology, Kyung Hee University Hospital, Kyung Hee University College of Medicine, #23 Kyungheedae-Ro, Dongdaemun-Gu, Seoul, 02447 Republic of Korea; 3 Department of Medicine, Graduate School, Kyung Hee University, #23 Kyungheedae-Ro, Dongdaemun-Gu, Seoul, 02447 Republic of Korea

**Keywords:** Metastasis, Computational models, Machine learning, Image processing, Cancer imaging

## Abstract

Brain metastases (BM) are the most common intracranial tumors, and their prevalence is increasing. High-resolution black-blood (BB) imaging was used to complement the conventional contrast-enhanced 3D gradient-echo imaging to detect BM. In this study, we propose an efficient deep learning algorithm (DLA) for BM detection in BB imaging with contrast enhancement scans, and assess the efficacy of an automatic detection algorithm for BM. A total of 113 BM participants with 585 metastases were included in the training cohort for five-fold cross-validation. The You Only Look Once (YOLO) V2 network was trained with 3D BB sampling perfection with application-optimized contrasts using different flip angle evolution (SPACE) images to investigate the BM detection. For the observer performance, two board-certified radiologists and two second-year radiology residents detected the BM and recorded the reading time. For the training cohort, the overall performance of the five-fold cross-validation was 87.95%, 24.82%, 19.35%, 14.48, and 18.40 for sensitivity, precision, F1-Score, the false positive average for the BM dataset, and the false positive average for the normal individual dataset, respectively. For the comparison of reading time with and without DLA, the average reading time was reduced by 20.86% in the range of 15.22–25.77%. The proposed method has the potential to detect BM with a high sensitivity and has a limited number of false positives using BB imaging.

## Introduction

Brain metastases (BM) are the most common intracranial tumors and commonly originate from lung cancer, breast cancer, and malignant melanoma^1^. Its prevalence has been increasing because of the prolonged survival of cancer patients following improvements in systemic treatment options^2,3^ and improved lung cancer screening programs in many countries^4–6^. The contrast-enhanced T1-weighted imaging (CE T1WI) magnetic resonance (MR) sequences are key in the diagnosis of BM and are also used for longitudinal follow-up to assess the treatment response. Most patients present with three or fewer metastases in the brain; however, 40% of the patients have more number of metastases^7^. Detection of the presence of metastases in the initial work-up of tumor patients, delineation of the initial tumor volume, and volume changes in relation to therapy are key tasks for radiologists, although the identification of BM is a time-consuming and tedious manual process for radiologists^8^. The presence of BM can alter the overall oncologic management; hence, early and accurate diagnosis of BM is crucial for appropriate treatment planning.

Recently, deep learning-based approaches have been proposed to assist radiologists by automatically detecting or segmenting BM on CE T1WI^9–11^. However, it is also a challenging task because of the similar morphological properties of BM and other structures, such as the intracranial vessels, as well as large variations in the size and distribution of BM. Recently, Grovik et al. presented an automatic detection and segmentation algorithm using multi-sequence MRI to overcome the limitations of using only one sequence of CE T1WI^12^. From this point of view, the accurate detection of BM and their differentiation from different suspicious regions (BM mimics) are important for appropriate diagnosis and treatment.

Black-blood (BB) imaging is used to complement the contrast-enhanced 3D gradient-echo (CE 3D GRE) imaging to detect BM, wherein variable refocusing flip angles combined with flow-sensitizing gradients are used, selectively suppressing moving blood while stationary tumor contrast remains visible^13^. These sequences can be useful in acquiring 3D brain data from BM patients and overcoming the disadvantages of the 3D magnetization-prepared rapid gradient echo (MPRAGE), which was originally commonly used for detecting BM^13,14^ because of its high spatial resolution and low partial volume effects. Single-slab 3D turbo spin echo BB images with slab selective, variable excitation pulses, such as sampling perfection with application-optimized contrasts using different flip-angle evolution (SPACE), enable the acquisition of high-resolution 3D datasets with contrasts similar to those obtained from 2D T2-weighted, T1-weighted, proton density, and dark fluid protocols. Furthermore, to improve the signal-to-noise ratio (SNR), SPACE MRI was acquired using compress-sensing. It does not require further post-processing to minimize the noise because high SNR MR imaging has less noise^15,16^.

The aim of our study was to provide a feasible deep learning algorithm (DLA) for the BM detection framework using the 3D BB imaging with contrast enhancement datasets that focus on variable sizes and locations in the routine clinical field. To verify the feasibility of BB imaging for BM detection, the same DLA was developed using the 3D MPRAGE. Moreover, a comparison of reading times with and without DLA was performed to estimate the expectation of reducing the workload of radiologists or clinicians when a BM detection framework can be used as a screening application.

## Results

**Figure 1:**
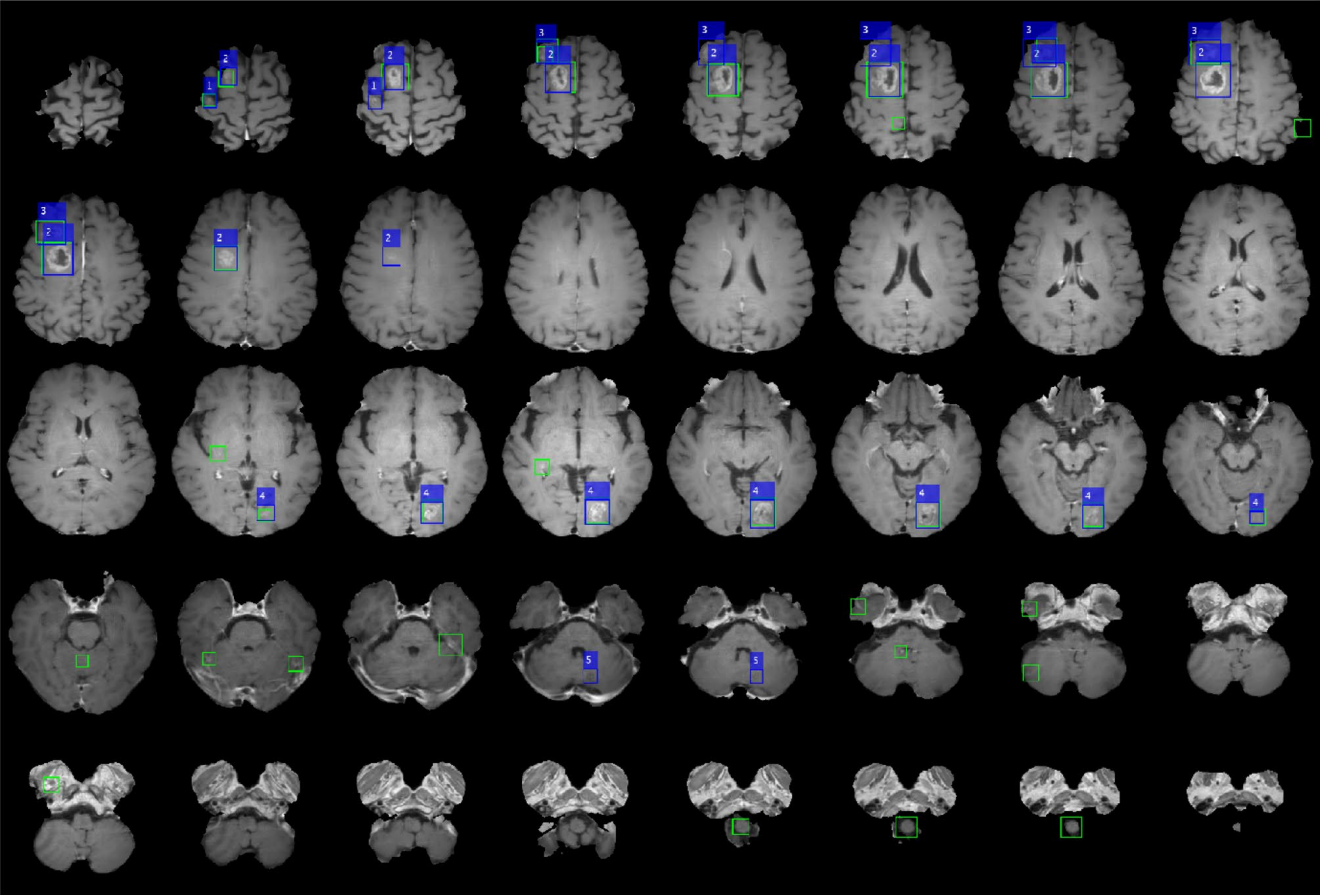
Example of a case of a 67-year-old male patient with five brain metastases. (Origin of metastases is non-small cell lung carcinoma) The numbers 1–5 are displayed at each lesion. Blue boxes show the label that the lesion placed more than two adjacent slices and green boxes show the prediction result by deep learning algorithm. This figure was generated by MATLAB (MathWorks, R2020b, Natick, MA, USA).

**Figure 2:**
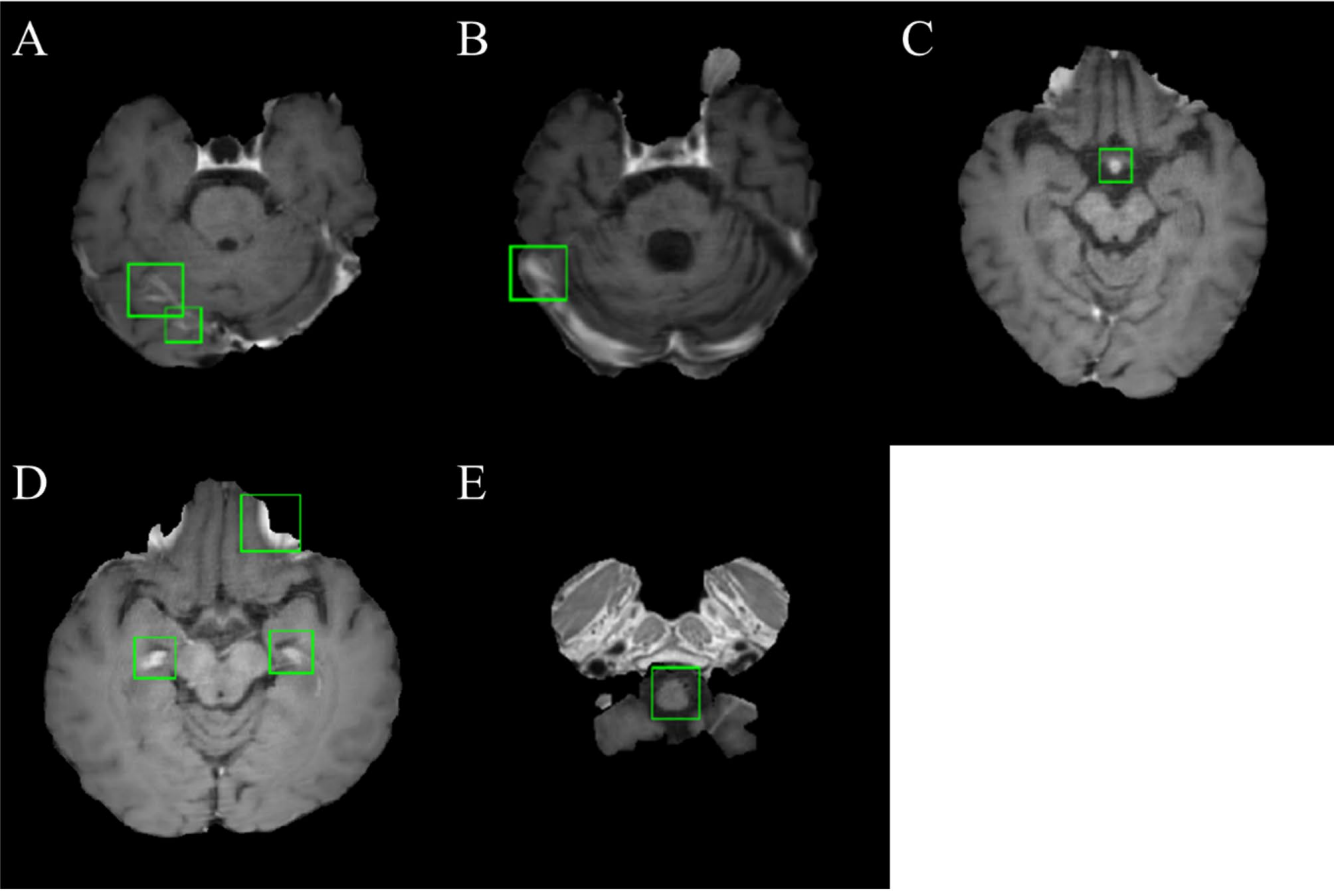
Examples of a typical false positive from the DLA. Sample of sampling perfection with application-optimized contrasts using different flip-angle evolution (SPACE) images demonstrated false positive regions around insufficiently suppressed vessel (**A**, **B**), basilar artery (**C**), choroid plexus (**D**), and medulla oblongata (**E**). False positives were overlaid with green boundary boxes. This figure was generated by MATLAB (MathWorks, R2020b, Natick, MA, USA).

An example of a patient with five metastatic lesions of non-small cell lung carcinoma is shown in Fig. 1. Table 1 shows the performance of the five-fold cross-validation of DLA. The overall performance was 87.95%, 24.82%, 19.35%, 14.48, and 18.40 for the sensitivity, precision, F1-Score, and false positive average (FP_avg_) for the training cohort and normal individual dataset, respectively. For five-fold cross validation, the maximum and minimum sensitivities were 97.44% (DataSet5) and 81.82% (DataSet3), respectively, and the maximum and minimum FP_avg_ were 21.57 (Model 2) and 7.27 (Model 3), respectively. Figure 2 shows examples of false positives from the DLA. The regions where false positives were mainly found were insufficiently suppressed in the vessel region. In addition, false positives were often observed in the choroid plexus, medulla oblongata, and basilar artery.

**Table 1:** Cross validation performances of DLA for BM detection using SPACE image.

	TPs	FNs	Sensitivity (%)	Precision (%)	F1-Score (%)	FP_avg_	FP_avg_Normal_
Overall performance	540	74	87.95	24.82	19.35	14.48	18.40
**Cross validations**							
DataSet1	142	20	87.65	23.39	18.47	21.14	31.22
DataSet2	147	23	86.47	22.86	18.08	21.57	23.97
DataSet3	81	18	81.82	33.61	23.82	7.27	8.07
DataSet4	94	11	89.52	29.84	22.38	9.61	13.00
DataSet5	76	2	97.44	20.54	16.96	12.78	15.72

*DLA* deep learning algorithm, *BM* brain metastases, *SPACE* sampling perfection with application-optimized contrasts using different flip-angle evolution, *TPs* true positives, *FNs* false negatives, *FP*_*avg*_ false positive average, *FP*_*avg_Normal*_ false positive average for normal individual dataset.

The performance of DLA using the MPRAGE is described in Supplementary Material 2. The overall performance was 62.42%, 20.83%, 31.23%, 12.96, and 10.47 for the sensitivity, precision, F1-Score, and FP_avg_ for the training cohort and normal individual dataset, respectively. The region where false positives were mainly found was the enhanced blood vessels including sinus that appeared as a bright region in the image, and the examples of the results of DLA using the MPRAGE images and the false positive examples are demonstrated in Supplementary Materials 3 and 4, respectively.

**Figure 3:**
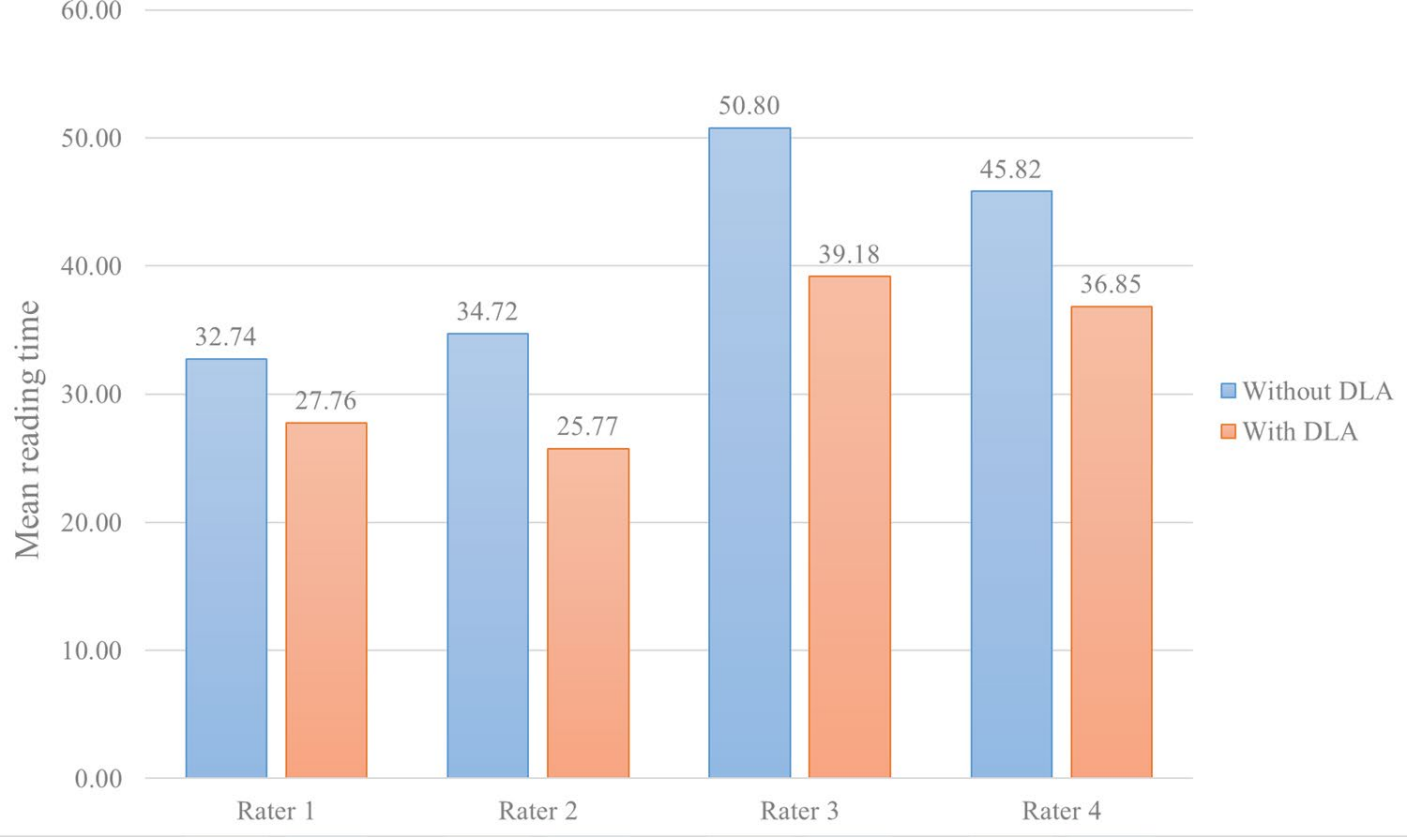
Comparison of the reading time for 20 individuals by four different raters with or without deep learning algorithm as computer-aided detection system. The bar graph represents the average of reading time [s] without and with deep learning algorithm.

To compare the time cost for the diagnosis of metastases, the reading time measurements with and without DLA as computer-aided detection were performed during the diagnosis by four radiologists for 20 individuals. Figure 3 presents the average reading times with and without DLA for the four radiologists. A decrease in reading time with DLA was observed by all raters, and the percentages of reduction in reading time with DLA were 15.22%, 25.77%, 22.88%, and 19.57% for the four radiologists. In addition, we also compared the reading time with and without DLA using a paired samples t-test (Supplementary Materials 5), and the reading time with DLA for all the radiologists showed a significant decrease (P < 0.0001). For the DLA, the average processing time was measured at 4.8 s.

## Discussions

In this study, a deep learning detection algorithm for BM using the contrast-enhanced BB imaging data was developed. Lesion candidates were automatically selected by presenting more than two slices. This type of computer-aided diagnostic system could be clinically beneficial because reducing the number of patients with possible tumors could help reduce the total time required for radiologist interpretation.

Compared with the CE 3D-GRE, which is commonly used for BM detection, BB imaging, which selectively suppresses moving blood, can be useful in suppressing contrast-enhanced vessels that can be recognized as false positives. Compared to the DLA using MPRAGE, high sensitivity was observed in the DLA using BB imaging; however, higher false positives were also observed. In terms of sensitivity, similar to these results, a previous study compared the performance of deep learning segmentation for BM on BB images and GRE. Park et al.^17^ reported that a deep learning model using the 3D BB images showed a higher sensitivity than a model using the 3D GRE images. In their study, the model using the 3D BB + 3D GRE showed the highest sensitivity; however, compared to the deep learning model using 3D BB + 3D GRE, a slightly lower sensitivity (0.5%) was observed in the deep learning model using 3D BB only. In terms of false positive, although both DLAs detected blood vessels as false positives, however, the DLA using the MPRAGE image showed a tendency to detect relatively fewer false positives in the region that the DLA using the BB image detected as false positives. Among them, the false positives of the insufficient suppressed transverse sinus decreased noticeably (Supplementary materials 5). The reason that the higher FP was measured in the DLA using BB images compared to the DLA using MPRAGE images is presumed to be the result of differences in the characteristics of the two datasets, while the deep learning model trains the data. In the case of BB imaging, the signals of most small blood vessels are suppressed, and there is a possibility that the DLA is biased toward detecting bright regions in the process of learning BM labels. On the other hand, both BM and vessels showed a bright signal in the MPRAGE image; however, only BM were labeled, and the vessels were not. Therefore, in the process of training the DLA using the MPRAGE images, it is possible that the distinction between vessels and BM showing bright signals was reinforced, and for this reason, low false positives may have been observed in DLA using the MPRAGE images. In this study, considering the clinical environment in which GRE images are not acquired as a routine, we developed a deep learning model using the BB images that expected better performance in sensitivity than GRE.

In recent years, advances in DLA have suggested the possibility of detecting and segmenting primary brain tumors^18,19^. Methods utilizing traditional image processing and machine learning techniques, such as template matching^20,21^ and level sets^22^, have been reported to produce promising results. In recent years, convolutional neural network (CNN)-based approaches have been used extensively in a variety of medical imaging analyses, which hold a great value for BM interpretation. However, only a few studies have applied such deep learning approaches in patients with BM, which may require different approaches, given their size and multiplicity^9,12,23^. Two previous studies used a deep learning model based on a CNN to detect BM. Zhou et al.^24^ investigated a single-shot detector to detect BM. They trained a network using only CE T1WI, reporting a sensitivity of 81% and a precision of 36%. Compared to the previous study, the proposed method presented a contradictory overall performance, with higher sensitivity and lower precision. Zhang et al.^25^ investigated deep learning networks with detection methods using various MRI sequences, such as spoiled gradient-recalled (SPGR), MPRAGE, SPACE, and volumetric interpolated breath-hold examination (VIBE). They focused on false-positive reduction using the random undersampling boosting (RUSBoost) method, and reported a sensitivity of 87.10 and an FP_avg_ of 19 per scan. Compared with previous studies using the four-channel data as input, the proposed method showed similar sensitivity and lower FP_avg_ using a single sequence without any additional processing.

Previous studies used a deep learning model-based segmentation method to detect BM, and Charron et al.^23^ investigated using the DeepMedic neural network for detecting and segmenting BM in a multi-sequence MR database, including CE T1WI, T2-weighted imaging (T2WI), fluid attenuated inversion recovery (FLAIR), and T1WI. They focused on parameter optimization, and the results of their study showed 93% accuracy and 7.8 FP per patient. More recently, Grovik et al.^12^ used GoogLeNet-based CNN for BM detection with multiple MRI sequences for each patient: T1WI 3D fast spin-echo (CUBE), CE T1WI 3D axial inversion recovery prepped fast spoiled gradient echo (IR-prepped FSPGE), and 3D CUBE FLAIR. They reported 83% sensitivity and an FP rate of 8.3. The segmentation method used in previous studies has the advantage of taking a margin of the BM for treatment planning; however, labeling for the ground truth requires an enormous amount of manpower and time. The detection method, which was the same as our approach, could reduce the manpower and time consumption and achieve a performance similar to that of the segmentation method.

To apply this clinically, the verification of normal data is essential. The normal dataset that does not have a BM can evaluate FP_avg_ only, and the performance of FP_avg_ was 18.40, which is higher than the FP_avg_ for the training cohort. This result is assumed to be the reason for the pre-processing normalization step. The deep learning network is trained with labels pointing to the enhanced BM region. The cortical region of the normal dataset that was not enhanced could have a higher intensity than that of the BM dataset following the normalization processing. Thus, a deep learning model that is not trained with normal brain images misunderstands the normal cortical regions as false positives. The other cause of the high FP in our study was the normal sinus system, which had a high signal intensity on BB imaging. The proposed DLA detects false positives for all healthy data sets, and further studies to reduce false positives to at least one or less should be performed for a deep learning network to be used as screening software.

For the comparison of reading time with and without DLA, four radiologists showed a decrease in reading time with DLA, and the average reading time decreased by 20.86% in the range of 15.22–25.77%. Considering that the DLA in this study presented a false positive of 14.48 for BM patient and 18.40 for normal individuals on average, further reduction of reading time can be expected if a DLA with higher sensitivity and lower false positives is developed. There was no significant difference between the board-certified radiologists and radiology residents in the reading time reduction of BM detection with and without the DLA (P = 0.449).

Our study has several limitations. First, most diagnoses of BM are based on radiologic findings without pathologic confirmation, because multiple small metastatic lesions are usually not resected in clinical practice. Thus, we could not completely eliminate the possibility of false positives; however, we thoroughly reviewed both the initial and follow-up MRI scans. Second, to apply the deep learning model in real clinics, generalizability must be validated before distributing the deep learning model as an assistant software. Another limitation of this study was that the images were obtained at the single institution using an MR scanner from the same vendor with the same acquisition protocols for efficient training, which may not ensure the generalizability of deep learning. To suggest practical applicability, we are preparing further studies that include false-positive reduction, validation of generalizability via external validation, and evaluation of the time consumption of radiologists with or without deep learning algorithms. Third, although BM lesions with a diameter < 5 mm were excluded from the response assessment criteria for brain metastases, diagnosing and detecting small BM lesions remain a challenging issue. Therefore, we are preparing a further study to detect small BM by applying a deep learning model that specializes in small object detection. In this study, we provide a BM detection DLA for 3D BB imaging with contrast enhancement datasets. Although the performance of the proposed DLA is not sufficient for direct use as a screening application, further studies focused on reducing false positives, training on recognizing small BM, and verifying the generalizability via external validation. Our DLA method was developed to facilitate the daily routine work of radiologists by screening patients in advance, and helping improve diagnostic sensitivity because even experienced radiologists often miss BM^26^.

## Materials and methods

### Patient population

**Figure 4:**
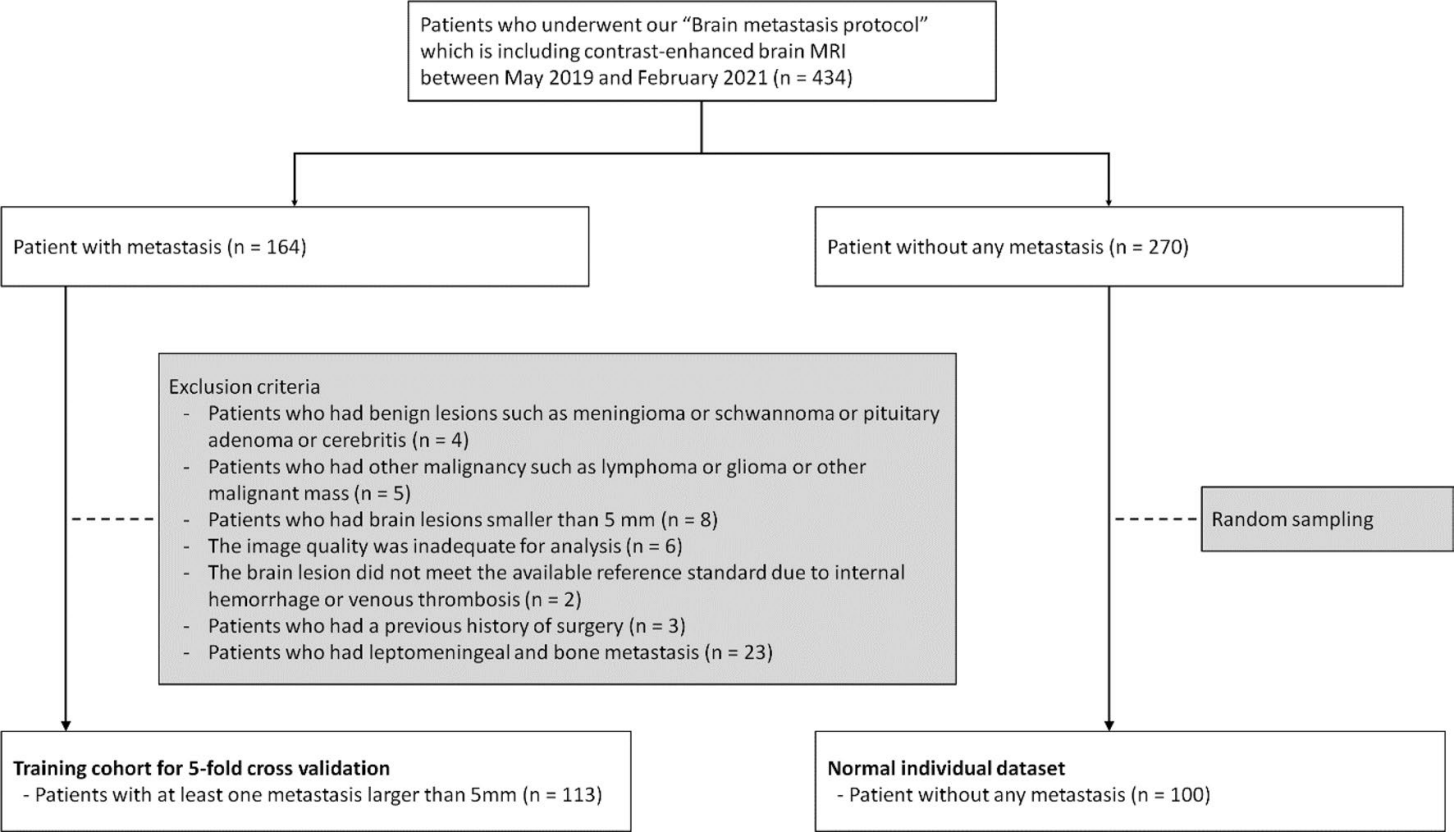
The flowchart of the study population.

This retrospective, single-center study was approved by the institutional review board of Kyung Hee University, and the requirement for informed consent was waived. The study population comprised a training cohort for five-fold cross-validation and a normal individual dataset (Fig. 4). The picture archiving communication system (PACS) and electronic medical records were retrospectively searched, and 434 patients who underwent our BM protocol, including contrast-enhanced brain MRI before treatment, were identified between May 2019 and February 2021. For the training cohort, 164 patients who were recently diagnosed with BM were selected, and a total of 51 patients were excluded for the following reasons: (1) four patients had benign lesions such as meningioma, schwannoma, pituitary adenoma, or cerebritis; (2) five patients had other malignancies such as lymphoma, glioma, or other malignant masses; (3) eight patients had brain lesions smaller than 5 mm; (4) the image quality in six patients was inadequate for analysis; (5) the brain lesion in two patients did not meet the available reference standard because of internal hemorrhage or venous thrombosis; (6) three patients had a previous history of surgery; and (7) 23 patients had leptomeningeal and bone metastases. Subsequently, 113 patients (6196 slices MR images in the BB axial image) with 585 metastases (1055 images) were included. Most cases inevitably require pathologic confirmation; thus, typical MRI findings and imaging follow-up for a minimum of 6 months were used to characterize BM. The mean patient age was 64.7 years (range 21–85 years). Primary malignancies included lung (n = 83), breast (n = 4), melanoma (n = 6), ovary (n = 1), gastrointestinal (n = 7), and miscellaneous (n = 12) cancers.

The training cohort was divided for five-fold cross-validation; the details of these datasets are listed in Table 2. For the healthy individual dataset, 270 patients without BM were identified. Among them, 100 patients were selected in the study using random sampling after data cleaning, such as excluding missing data. The mean age of the healthy individual dataset was 68.3 years for 66 men and 34 women.

**Table 2:** Characteristics of the datasets.

	Training cohort						Normal dataset
	Total	Fold 1	Fold 2	Fold 3	Fold 4	Fold 5	
No. of MRI scans	113	22	23	22	23	23	100
No. of slices	6196	1205	1260	1206	1265	1260	5452
No. of lesion	585	163	171	68	105	78	
No. of slices with lesion	1055	279	175	165	234	202	
Age [years] (mean ± std)	65 ± 11.3	65.8 ± 12.1	68.6 ± 7.4	61.9 ± 16.4	64.6 ± 7.5	64.2 ± 9.7	68.3 ± 10.9
**Sex**							
Male	70	14	16	15	13	12	66
Female	43	8	7	7	10	11	34
**Origin (per scan)**							
Lung	83	15	22	16	18	12	
Breast	4	2	0	0	0	2	
Melanoma	6	3	0	1	0	2	
Ovary	1	0	0	0	1	0	
Gastrointestinal	7	1	1	2	1	2	
Miscellaneous	12	1	0	3	3	5	

*MRI* magnetic resonance imaging.

### Image acquisition

Brain MRI (3 T MAGNETOM VIDA; Siemens, Erlangen, Germany) was performed in patients with underlying primary malignancies. The MRI protocol for BM included post-contrast 3D BB images (SPACE). The imaging parameters were as follows: repetition time, 700 ms; echo time, ms; slice thickness, 0.8 mm; flip angle, 120°; matrix size, 288 × 288; field of view, 230 × 230 mm^2^; voxel size, 0.8 × 0.8 mm^2^. After acquiring the 3D BB images in the sagittal plane, the image reconstruction in the axial plane was performed using the following parameters: slice thickness, 3 mm; matrix size, 512 × 512; pixel size, 0.45 × 0.45 mm^2^. For gadolinium (Gd)-enhanced imaging, a dose of 0.1 mmol/kg body weight of gadobenate dimeglumine (MultiHance, Bracco Diagnostics, Princeton, NJ) was intravenously administered.

### Data labeling

To propose an automatic lesion detection DLA, a post-contrast BB image with 3D axial reconstruction was used for the training. Two neuroradiologists with 10 and 30 years of experience, respectively, detected all the metastases on the post-contrast BB axial images of the patients included in the training cohort on the PACS, and a rectangular region of interest bounding each lesion was drawn using the Image Labeler application included in the MATLAB program (MathWorks, R2020b, Natick, MA, USA).

**Figure 5:**
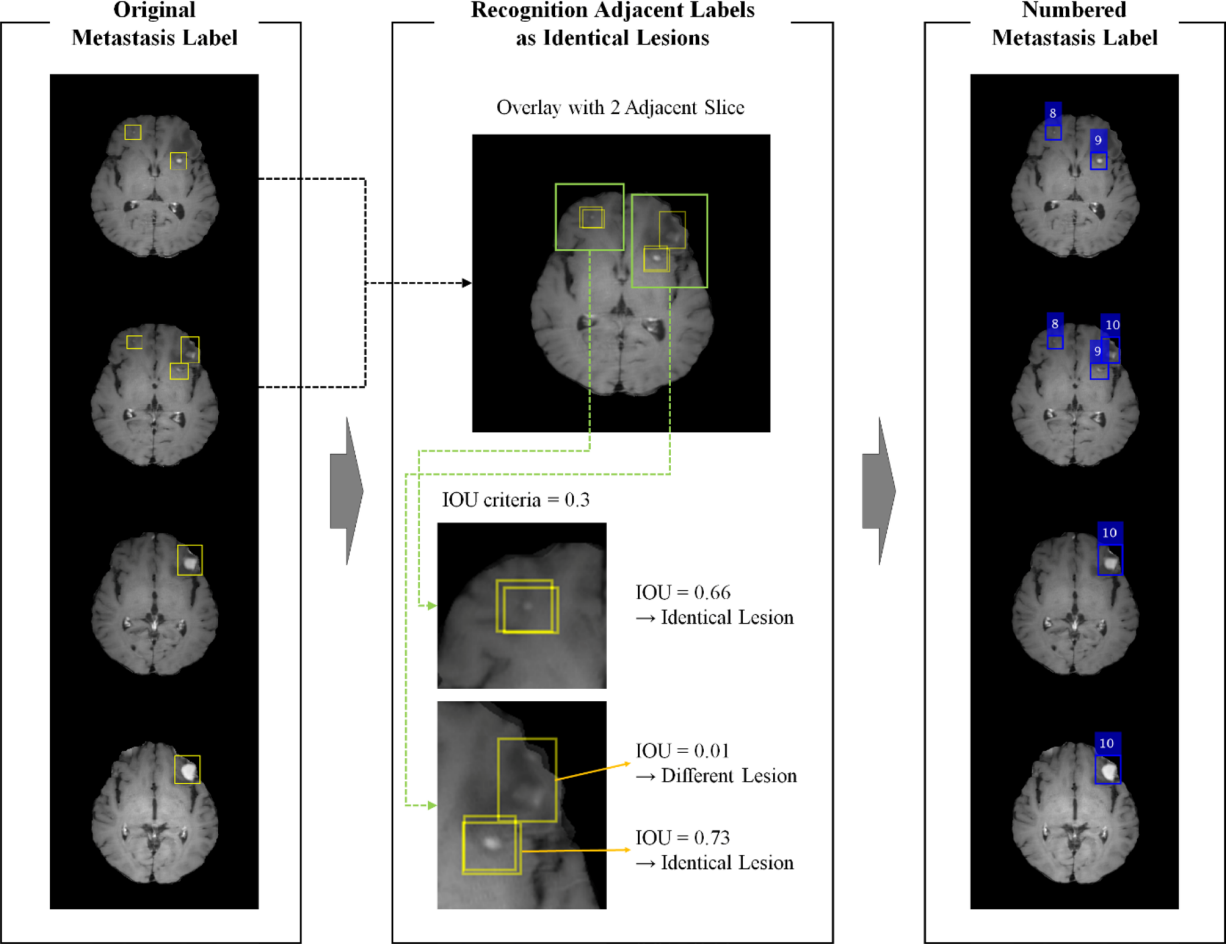
The flowchart for recognition of adjacent labels as identical lesions. Each label was identified per lesion and granted the lesion number for each lesion. Yellow boxes show the original labels and blue boxes show the label that the lesion placed more than two adjacent slices. This figure was generated by MATLAB (MathWorks, R2020b, Natick, MA, USA) and PowerPoint 2016 (www.microsoft.com).

Existing object detection models provide predictions for each slice. However, clinically, the provision of predictions for each lesion is more practical. Thus, the DLA was designed to automatically derive the predictions for each lesion. For all labels on adjacent slices, the labels with an intersection > 0.3 intersection over union (IoU) were recognized as lesions, and each lesion was assigned an independent number (Fig. 5). As the DLA was designed for the detection of BM with a diameter > 5 mm that should be observed over two or more slices with a slice thickness of 3 mm, labels in a single slice were excluded for training and evaluation.

### Training the proposed DLA

**Figure 6:**
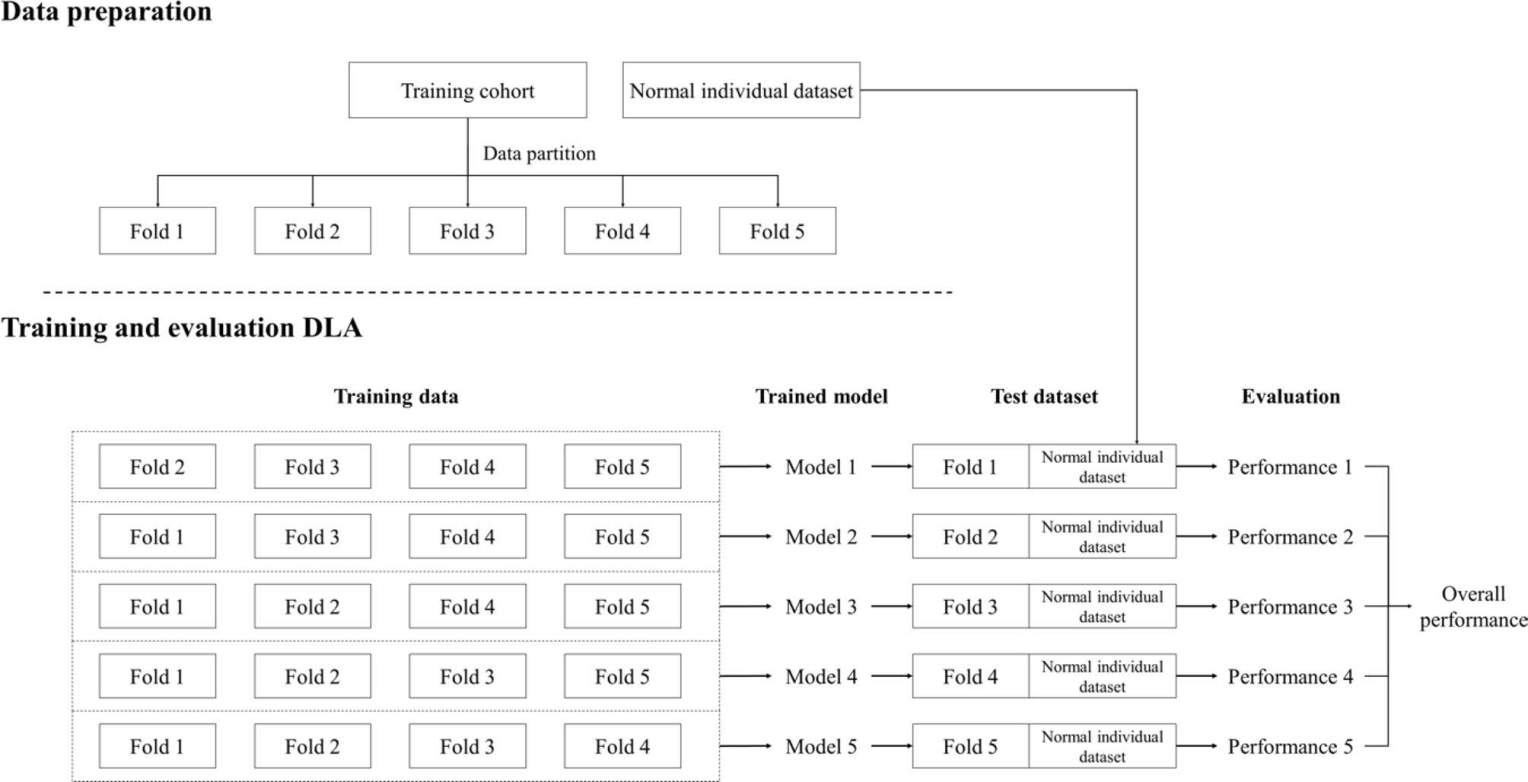
Five-fold cross-validation diagram using training cohort dataset and normal individual dataset.

In this study, five-fold cross-validation was performed to overcome the lack of a BM dataset. A diagram of five-fold cross-validation for the training cohort and the normal individual dataset is illustrated in Fig. 6. Skull stripping was performed using a brain extraction tool (BET, v1.3)^27^ for all training cohort data and healthy individual datasets before use. After skull stripping, image intensity normalization in the range of 0–1 was performed for each slice.

You Only Look Once (YOLO) V2^28^, a state-of-the-art CNN object detection algorithm that can simultaneously detect the locations of objects in input images and classify them into different categories, was used for the DLA architecture. A YOLO V2 network for each five-fold cross-validation was initialized using the transfer learning method based on pre-trained ResNet-50^29^, similar to that in a previous study^30^, with the following parameters: seven anchor boxes, Adam optimizer, mini-batch size of 64, initial learning rate of 1 × 10^−3^, factor for L2 regularization of 1 × 10^−4^, and 1,000 epochs at maximum. To compensate for the lack of training data, random image rotations (0°, 90°, 180°, and 270°) and left–right flip processing were implemented. Every single image with BM was used as input data for the YOLO V2 network, and the mean and total training time for five-fold cross-validation were 11 h and 30 min, and 57 h and 31 min, respectively. To investigate whether the BB imaging is more efficient than the MPRAGE in DLA for BM detection, the same DLA was trained using the MPRAGE; the detailed process is described in Supplementary Material 1.

All processes were performed on a single-server computer running a Windows operating system (Windows Server 2016) with a double NVIDIA V100 GPU and 32 GB of memory (Nvidia Corporation). The image labeling, processing, and training networks were based on MATLAB (MathWorks, R2020b, Natick, MA, USA).

### Performance evaluation of DLA

To evaluate the BM detection performance, test sets of five-fold cross-validation were evaluated by each trained model, with sensitivity, precision, F1-Score, and FP_avg_ as follows:

$$Sensitivity \left(TPR\right)=\frac{TP}{TP+FN}$$

$$Precision \left(PPV\right)= \frac{TP}{TP+FP}$$

$$F1-scores=2\times \frac{TPR\times PPV}{TPR+PPV}= \frac{2\times TP}{\left(2\times TP\right)+FN+FP}$$

$$F{P}_{avg}=\frac{FP}{N}$$

where TP is the true positive, FP is the false positive, FN is the false negative, TPR is the true positive rate, PPV is the positive predictive value, and N is the number of individuals. For all evaluations, the TP was determined when the IoU between the predicted box and the ground truth was > 0.5.

The purpose of the DLA is to screen for any pathology before a radiologist makes a diagnosis, and not to compare the DLA and humans. Thus, we assumed that the detection of a part of the lesion can assist radiologists, and predicted results using DLA that only one slice of the whole lesion estimates as true positive.

To evaluate how the trained network predicts an individual without BM, a healthy individual dataset was evaluated using all five networks from a five-fold cross-validation, and the results were averaged. The normal individual dataset had no ground-truth label data, and only FP_avg_ was calculated.

### Observer performance

Observer performance tests for measuring reading time with and without DLA were conducted to estimate the expectation of reducing the workload of radiologists for the BM detection framework. For the test datasets, 13 MRI scans with BM were randomly selected from the training cohort, and seven MRI scans without BM were randomly selected from the healthy individual datasets for this test.

The readers comprised two groups: two board-certified radiologists with 10 and 3 years of experience in neuroradiology (raters 1 and 2), and two second-year radiology residents (raters 3 and 4). The readers were informed that the BB imaging was performed for BM work-up but were not provided with information regarding the presence of BM or other clinicopathological histories. The readers performed BM detection with and without DLA with a time interval of more than 1 year (14 months), and recorded the reading time only on BB axial imaging with contrast enhancement.

### Institutional review board statement

This study was conducted according to the guidelines of the Declaration of Helsinki and was approved by the Ethics Committee of the medical faculty of the Kyung Hee University (KHU-2021-06-070).

## Supplementary Information

Supplementary Information.

## Data Availability

The datasets used in this study are available upon request from the corresponding author. The datasets are not publicly available because to the various patient information.
