# Supplementary material for: Deep learning-based detection algorithm for brain metastases on black blood imaging
Source: Sci Rep. 2022 Nov 14;12:19503. doi: 10.1038/s41598-022-23687-8 (PMC9663732; doi:10.1038/s41598-022-23687-8)
Supplement: Supplementary file 1 — Supplementary Information. [file 41598_2022_23687_MOESM1_ESM.docx]

**Deep Learning-Based Detection Algorithm for Brain Metastases on Black Blood Imaging**

Jang-Hoon Oh^1^, MS, Kyung Mi Lee^2*^, MD, PhD, Hyug-Gi Kim^2^, PhD, Jeong Taek Yoon^3^, MD and Eui Jong Kim^2^, MD, PhD

**Supplementary Materials**

Supplementary Materials 1. Developing the Deep Learning Algorithm using MPRAGE Images

To verify the feasibility of DLA using BB images, contrast-enhanced magnetization prepared rapid gradient echo (MPRAGE) images were acquired using a 3T MRI system (3T MAGNETOM VIDA, Siemens, Erlangen, Germany). The imaging parameters for CE MRPAGE were: repetition time = 8.1 ms; echo time = 3.7 ms; flip angle = 8°; slice thickness = 0.8 mm; matrix size = 288 × 288; field of view = 236 × 236 mm^2^; and voxel size = 1 × 1 × 1 mm^3^. After acquire the 3D BB images in sagittal plane, image reconstruction in axial plane was performed same as sampling perfection with application-optimized contrasts using different flip angle evolution (SPACE) image with the parameters as follows: slice thickness, 3 mm; matrix size, 512 × 512; pixel size, 0.45 × 0.45 mm^2^.

The cohort dataset same as the deep learning algorithm(DLA) for SPACE were used to train and evaluate the DLA for MPRAGE, but only one individual in the fourth cross-validation fold was missed. the other processing was performed the same as developing and evaluating the DLA for SPACE.

Supplementary Materials 2. Cross validation performance of DLA using MPRAGE

|  | TPs | FNs | Sensitivity | Precision | F1-Score | FP_avg_ | FP_avg_Normal_ |
| --- | --- | --- | --- | --- | --- | --- | --- |
| Average | 382 | 230 | 62.42% | 20.83% | 31.23% | 12.96 | 10.47 |
| CrossValidations |  |  |  |  |  |  |  |
| DataSet1 | 88 | 75 | 53.99% | 21.41% | 30.66% | 14.68 | 9.41 |
| DataSet2 | 103 | 68 | 60.23% | 33.44% | 43.01% | 8.91 | 6.93 |
| DataSet3 | 51 | 48 | 51.52% | 14.57% | 22.72% | 13.59 | 11.76 |
| DataSet4 | 78 | 23 | 77.23% | 14.00% | 23.71% | 21.77 | 20.10 |
| DataSet5 | 62 | 16 | 79.49% | 29.81% | 43.36% | 6.35 | 4.16 |

Supplementary Materials 3. Example result of DLA using MPRAGE image


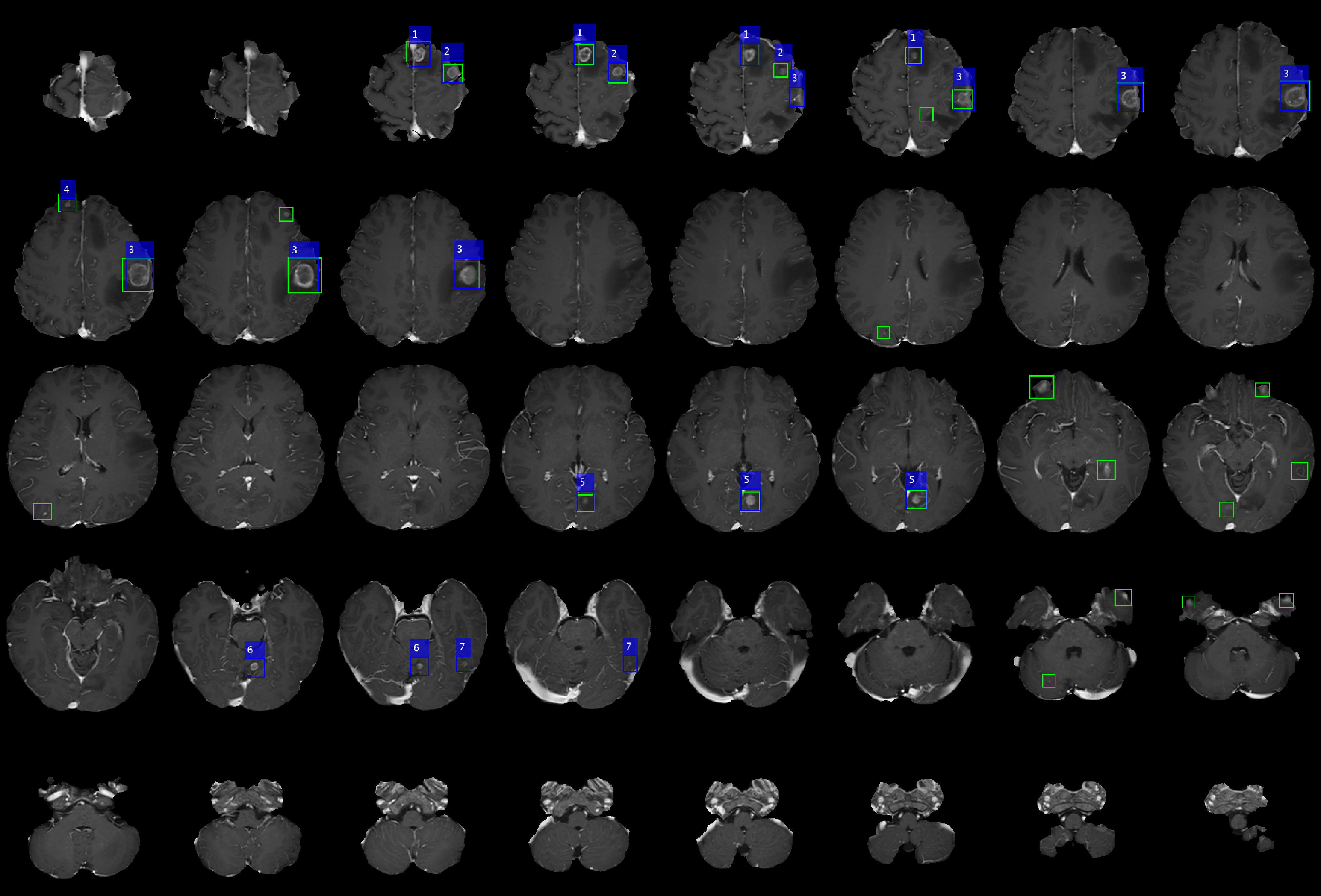


Supplementary Materials 4. False positive examples of the DLA using MPRAGE image. The DLA using MPRAGE image predicted the enhanced blood vessels including sinus. All false positives are represented with green boundary boxes


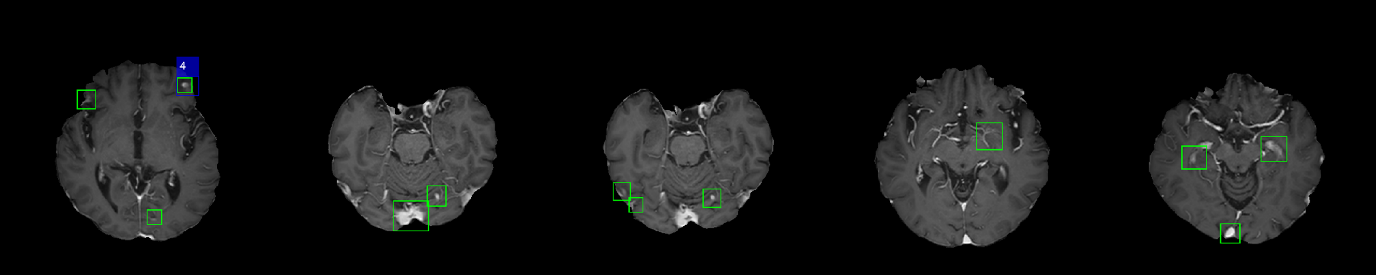


Supplementary Materials 5. Examples in which FP detected in DLA using the BB image was not detected in DLA using the MPRAGE image. The ground truth and predicted box are marked with blue and green boxes, respectively. The FPs around insufficient suppressed vessel in SPACE GD image, such as transverse sinus, were not detected as FP from DLA using the MPRAGE image.


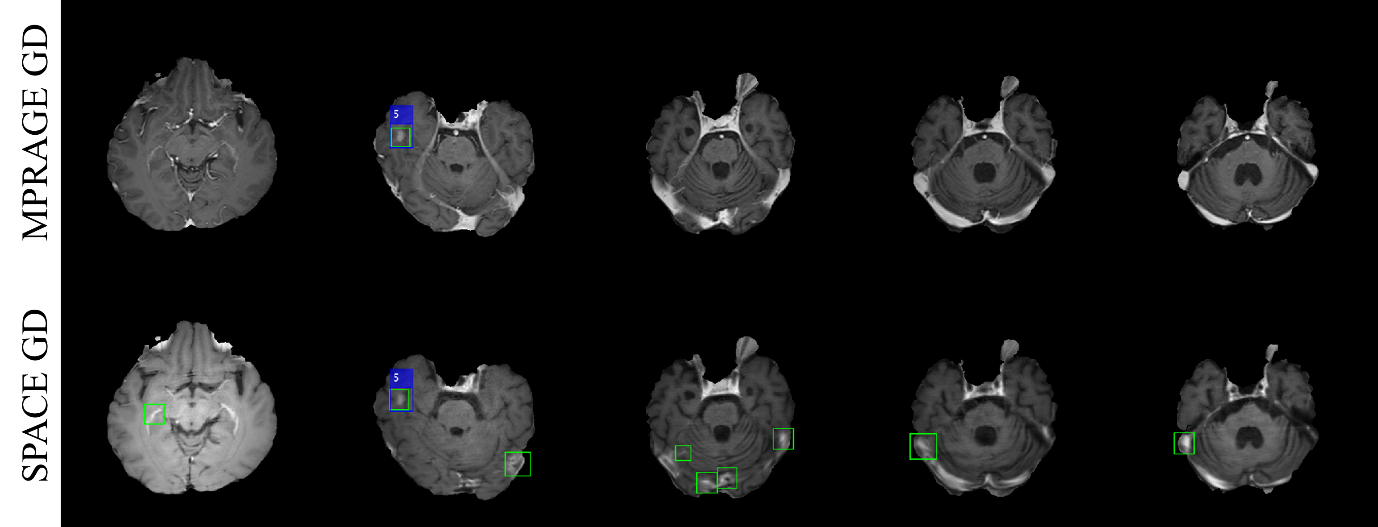


Supplementary Materials 6. Result of a paired samples t-test for reading time with and without DLA.

|  | **Without DLA** | | |  | **With DLA** | | |  | **Probability** |
| --- | --- | --- | --- | --- | --- | --- | --- | --- | --- |
|  | **Mean** | **95% CI** | **SD** |  | **Mean** | **95% CI** | **SD** |  |  |
| Overall performance | 41.02 | 37.23 to 44.81 | 17.04 |  | 32.39 | 29.33 to 35.45 | 13.75 |  | P < 0.0001 |
| Rater 1 | 32.74 | 28.33 to 37.16 | 9.43 |  | 27.76 | 23.96 to 31.56 | 8.12 |  | P < 0.0001 |
| Rater 2 | 34.72 | 32.01 to 37.44 | 5.80 |  | 25.77 | 23.53 to 28.02 | 4.80 |  | P < 0.0001 |
| Rater 3 | 50.80 | 39.83 to 61.77 | 23.44 |  | 39.18 | 30.45 to 47.91 | 18.66 |  | P < 0.0001 |
| Rater 4 | 45.82 | 37.75 to 53.88 | 17.22 |  | 36.85 | 30.05 to 43.66 | 14.54 |  | P < 0.0001 |
